# Ecotoxicological Estimation of 4-Cumylphenol, 4-*t*-Octylphenol, Nonylphenol, and Volatile Leachate Phenol Degradation by the Microscopic Fungus *Umbelopsis isabellina* Using a Battery of Biotests

**DOI:** 10.3390/ijerph19074093

**Published:** 2022-03-30

**Authors:** Tomasz Janicki, Andrzej Długoński, Aleksandra Felczak, Jerzy Długoński, Mariusz Krupiński

**Affiliations:** 1 Department of Industrial Microbiology and Biotechnology, Faculty of Biology and Environmental Protection, University of Lodz, Banacha 12/16 Street, 90-237 Lodz, Poland; tomaszjanicki@outlook.com (T.J.); aleksandra.felczak@biol.uni.lodz.pl (A.F.); jerzy.dlugonski@biol.uni.lodz.pl (J.D.); 2 Institute of Biological Sciences, Faculty of Biology and Environmental Sciences, Cardinal Stefan Wyszyński University in Warsaw, 1/3 Wóycickiego Street, 01-038 Warsaw, Poland; andrzej.dlugonski@biol.uni.lodz.pl; 3 Institute of Ecology and Environmental Protection, Faculty of Biology and Environmental Protection, University of Lodz, Banacha 12/16 Street, 90-237 Lodz, Poland

**Keywords:** biodegradation, detoxification, landfill post-industrial waste, battery of biotests, *Umbelopsis isabellina*

## Abstract

The phenolic xenobiotics nonylphenol (NP), 4-*tert*-octylphenol (4-*t*-OP), and 4-cumylphenol (4-CP) have the potential to seriously disrupt the endocrine system. Volatile phenols (VPs), especially those present in landfill leachate, also adversely affect the health of numerous organisms. Microbial degradation of xenobiotics can result in the formation of intermediates with higher toxicity than the precursor substrates. Therefore, the main aim of this study was to assess the changes in environmental ecotoxicity during the biotransformation of nonylphenol, 4-*tert*-octylphenol, 4-cumylphenol and volatile phenols by *Umbelopsis isabellina* using a battery of biotests. The application of bioindicators belonging to different taxonomic groups and diverse trophic levels (producers, consumers, and reducers) indicated a significant reduction in toxicity during the cultivation of fungus cultures both for nonylphenol, 4-*tert*-octylphenol, 4-cumylphenol and volatile phenols. The rate of toxicity decline was correlated with the degree of xenobiotic biotransformation. Removal of 4-cumylphenol and 4-*tert*-octylphenol also led to a decrease in the anti-androgenic potential. Moreover, this is the first report demonstrating the anti-androgenic properties of 4-cumylphenol. The results showed that *U. isabellina* is an attractive tool for the bioremediation and detoxification of contaminated environments.

## 1. Introduction

Many phenolic xenobiotics are commonly found in a variety of environmental matrices due to their wide industrial and household application. They are mainly applied as raw materials for the synthesis of chemical substances that possess rich technological and industrial properties and are used in the production, e.g., washing powders, cosmetics, plastic products, paints, varnishes, textiles, paper products, dyes, pesticides, pharmaceuticals [1,2]. Anthropogenic activities result in the continuous release of phenolic xenobiotics into aquatic and terrestrial habitats. The presence of these compounds was detected in many environments such as soil, groundwater, sediments, rivers, lakes, and sewage [2,3,4]. Among phenolic xenobiotics, nonylphenol (NP), 4-*tert*-octylphenol (4-*t*-OP), 4-cumylphenol (4-CP), and volatile phenols (VPs) seriously threaten the biodiversity and function of ecosystems. The adverse effects of 4-CP, NP, 4-*t*-OP, VPs on the reproduction, growth, development, and homeostasis of different organisms have been reported in several studies [5,6,7]. During the microbial degradation of both natural wastes and organic xenobiotics, toxic VPs are formed. These phenols pollute various environmental compartments such as ground and surface water or agricultural lands because they are often a harmful component of leachate from municipal and industrial landfills [5,8].

It has been recognized that 4-CP, NP, and 4-*t*-OP interfere with the normal functioning of the endocrine system; therefore, these compounds were included in the categories of endocrine-disrupting chemicals (EDCs) [6,9,10]. Because of the bioaccumulation capacity of 4-CP, NP, and 4-*t*-OP, their resistance to degradation and potential negative effects for both humans and wildlife, increasing attention has been given to developing effective methods for their decomposition and detoxification.

Microbial degradation is regarded as an important tool for the elimination and detoxification of xenobiotics. To estimate the biodegradation efficiency, evaluation of the toxicity reduction is a more appropriate and reliable approach than only measuring the residual concentration of the test compound during its biotransformation processes. There are many cases where microbiological degradation of xenobiotics leads to the formation of intermediates characterized by higher toxicity than that of the precursor compound [11,12]. Moreover, antagonistic and synergistic effects due to unknown metabolite interactions may also result in enhanced toxicity. Therefore, from an ecotoxicological perspective, it is very important to use bioassays that allow for a complete evaluation of the toxicity of products resulting from biodegradation processes and prove exact information about their potential environmental impact.

Many biological tests using different organisms as bioindicators have been effectively applied to examine the ecotoxicity of various organic contaminants [13,14,15]. Bioindicators allow for relevant information about the biological effects of pollutants on ecosystem structure and function to be obtained.

The use of single bioassays to estimate toxicity changes in xenobiotic biodegradation processes has been reported in several studies [14,16,17,18]. However, there is little information about the toxicity variation during the microbial biodegradation of VPs and phenolic EDCs, particularly 4-CP, 4-*t*-OP, and NP. In our previous paper, we demonstrated the ability of the fungus *Umbelopsis isabellina* to efficiently degrade the phenolic xenobiotics 4-CP, 4-*t*-OP, and NP [19]. By using assays with two bioindicators belonging to consumers in the trophic chain (*Daphnia magna* and *Artemia franciscana*), we have also shown that the biodegradation processes lead to toxicity reduction. The sensitivity of organisms to contaminants varies widely; therefore, the use of a battery of bioassays involving bioindicators representing different trophic levels is an effective and required tool for predicting comprehensive environmental risk [12,20]. Studies concerning a complex assessment of the total hazard posed by all the intermediates formed during microbial bioconversion by using bioindicators from different taxonomic groups remain limited. To our knowledge, the assessment of endocrine activity in the course of microbial degradation of phenolic EDCs has been rarely analyzed. Therefore, there is a need to find microbial strains capable of growing and metabolizing in the presence of toxic pollutants and estimate the toxicological effects of intermediates along with precursor contaminants to specify the efficiency of detoxification as a result of biodegradation mechanisms.

In this context, the main aim of this study was to assess the toxicity removal efficiency during alkylphenol (4-CP, NP and 4-*t*-OP) degradation by the fungus *U. isabellina* by using selected bioassays covering multiple toxicological endpoints. In the present work, a multispecies toxicological approach was applied by using a battery of biotests containing organisms belonging to different trophic levels and exhibiting different key functions in the ecosystem: decomposers, producers, and consumers. Additionally, the endocrine properties, including anti-androgenic activity, of selected phenols were also determined. In the face of increasing environmental pollution with leachates generated from post-industrial landfills and containing various toxic phenols, this work also focused on determining the possibility of using the tested strain to remove and detoxify VPs from leachates loaded with complex organic contaminants. Thus, for the first time, studies were conducted to assess the ability of a single, non-ligninolytic fungal strain to eliminate and detoxify phenolic compounds, including EDCs, both from cultures with single xenobiotic supplementation and from multi-contaminated environmental matrices.

## 2. Materials and Methods

### 2.1. Chemicals

NP, 4-CP, and 4-*t*-OP were purchased from Sigma-Aldrich (St. Louis, MO, USA). Other solvents and reagents were of a highly pure grade and obtained from Merck (Darmstadt, Germany). All constituents of the microbial culture media were provided by Becton Dickinson (Heidelberg, Germany).

### 2.2. Landfill Leachate Preparation

Landfill leachate was collected from the closed dangerous waste landfill of the former “Boruta” Dye Factory in Zgierz, Poland (Figure S1) and kindly shared by the Voivodeship Inspectorate of Environmental Protection in Łódź, Poland. Leachate sampling was carried out in accordance with the principles contained in the accredited test procedure based on the ISO 5667-10 standard. The preservation, transport, and storage of the tested matrices were performed as specified in the ISO 5667-3:2018 standard. Landfill leachate was collected with a polyethylene (PE) scoop into tight (screwed) PE containers with a capacity of 5 L and transported at 4 °C to the laboratory, where it was stored in a refrigerator at the same temperature and then analyzed within 24 to 48 h. Landfill leachate quality measurements were performed at the Main Research Laboratory in Łódź commissioned by the Voivodeship Inspectorate of Environmental Protection in Łódź, Poland, and are presented in Tables S1 and S2.

### 2.3. Microorganism and Growth Conditions

The filamentous fungus *U. isabellina* IM 833 was obtained from the Culture Collection of the Department of Industrial Microbiology and Biotechnology, University of Łódź, Poland. Fungal spores from 10-day-old cultures on ZT slants [19] were used to inoculate 20 mL of Sabouraud medium in a 100 mL Erlenmeyer flask. Cultivation was conducted at 28 °C for 24 h on a rotary shaker (150 rpm). After 24 h of incubation, precultures were transferred to fresh medium (2 mL of fungal inoculum and 18 mL of Sabouraud medium), and then NP, 4-*t*-OP, or 4-CP (dissolved in 96% ethanol, 5 mg mL^−1^ stock solution) was aseptically added to the cultures (100 µL of the stock solution was added to 20 mL of culture to obtain a final xenobiotic concentration of 25 mg L^−1^ in the medium). Control samples containing the same volume of ethanol (abiotic and biotic controls) were also prepared. All cultures were cultivated at 28 °C for 24 h on a rotary shaker (150 rpm). For cultures containing 20% and 40% of the landfill leachate, the incubation process was carried out for 96 h.

### 2.4. Biomass Estimation

The dry fungal biomass was determined by filtering the whole culture through Sartorius filter membranes (0.2 µm pore size) (preweighed and predried). After filtration, the samples were dried to a constant weight at 100 °C. All analyzed samples were triplicated, and the results are expressed as g L^−1^.

### 2.5. Ecotoxicological Analysis

A series of biotests using bioindicators belonging to different taxonomic groups and diverse trophic levels were carried out to estimate the effectiveness of reducing toxicity by biodegradation processes (Table S3). No toxicity of the untreated fungal cultures (biotic control) was observed for all indicator species used.

#### 2.5.1. Microorganism-Based Bioassays

Acute toxicity assessment was carried out on the basis of the decrease in *Aliivibrio fischeri* (DSM 7151) luminescence after exposure to the tested post-culture filtrates. The studies were performed according to a standard procedure (ISO 11348-2) using the Microtox M 500 Analyser (Modern Water, New Castle, DE, USA). Luminescence was measured before introducing the test matrices and after 15 min of incubating bacteria with the samples. After the test, the percentage of bioluminescence inhibition was calculated as the EC50 value by applying MicrotoxOmni software (Modern Water, New Castle, DE, USA).

The antimicrobial activities of the fungal cultures containing xenobiotics and their degradation products were also evaluated using the multispecies microbial assay for risk assessment (MARA) bioassay according to a standard protocol (NCIMB Ltd., Scotland, UK). Biotests were performed in 96-well plates containing lyophilized microorganisms (ten bacterial strains and one yeast) (Table S3). The results were calculated using MARA software (ver. 3.5T) and presented as microbial toxic concentration (MTC) values.

Growth inhibition tests using the freshwater green alga *Raphidocelis subcapitata* (SAG 61.81) and saltwater algae *Phaeodactylum tricornutum* (SAG 1090-1a) were conducted according to the standard ISO 8692:2012 and ISO 10253:2016 methods, respectively. After 72 h of incubation, algal growth reduction was calculated based on the number of cells in the control versus the number of cells in the treated cultures, and the results were expressed as EC50 values.

#### 2.5.2. Plant Bioassays

The phytotoxicity assessment of post-culture filtrates was based on the germination and seedling growth of one monocotyledonous (*Sorghum saccharatum*) and two dicotyledonous (*Lepidium sativum* and *Sinapsis alba*) species using the Phytotoxkit biotest (MicroBioTests Inc., Gent, Belgium). The experiments were carried out according to the instructions provided by the manufacturer. The number of germinated seeds and root lengths of the tested plants were examined using GIMP image analysis software. Inhibition of seed germination (IG) and root growth (IR) was calculated using the following formula:

IG or IR = [(*A* − *B*)/*A*] × 100%

where *A* is the mean seed germination or root length in the control samples and *B* is the mean seed germination or root length in the test post-culture filtrate samples. To assess the overall germination capacity, the germination index (GI) was calculated by comparing the IG and IR values using the following formula:

GI = (Gs × Ls)/(Gc × Lc) × 100%

where Gs and Ls are the seed germination in percent and root elongation in mm for the tested samples, respectively, and Gc and Lc are the corresponding values for the control samples.

#### 2.5.3. Invertebrate-Based Bioassays

A commercial test kit Rapidtoxkit (MicroBioTests Inc., Gent, Belgium), was used to estimate the acute toxicity of post-culture filtrates with the freshwater crustacean *Thamnocephalus platyurus*. The study evaluated the filtration and food intake of crustaceans exposed to the toxic agents present in the test samples in comparison with a reference system. Based on the percent inhibition of food particle uptake (a value ≥30% indicates the presence of harmful substances), EC50 values were determined for the tested cultures. Toxicity levels of filtrates from fungal cultures supplemented with landfill leachate (20% and 40% by volume) were evaluated using Artoxkit M and Daphtoxkit F toxicity bioassays (MicroBioTests, Inc., Gent, Belgium). The results of the tests were expressed as LC50 values. Lethal concentrations were determined from the linear portion of each curve using regression analysis.

#### 2.5.4. Endocrine Activity Assay

The XenoScreen YES/YAS Endocrine Disruptor Assay was used to determine the estrogenic and androgenic agonistic/antagonistic activity of the tested xenobiotics and post-culture liquids. The tests were performed in accordance with the standard provided by Xenometrix (Allschwil, Switzerland).

### 2.6. Statistical Analyses

Each treatment and control group contained triplicate samples. All experimental data are expressed as the mean ± standard deviation (SD). Statistical comparisons of the results between the control and treated samples were performed using one-way ANOVA followed by Tukey’s test. Values of *p* ≤ 0.05 were considered to be statistically significant. All statistical analyses were performed with Microsoft Office Excel and STATISTICA 13.3 (Tibco Software Inc., Carlsband, CA, USA) software.

### 2.7. Spatial Analyses

Maps illustrating the location of the landfill and its elements were elaborated based on geographic information systems (GIS) in QuantumGIS (ver. 3.12) software (Mountain View, CA, USA) and OSMStandard by OpenStreetMap GIS portal with its spatial database.

## 3. Results and Discussion

### 3.1. Ecotoxicological Assessment of Biodegradation Processes

The biodegradation efficiency of the tested phenolic xenobiotics to reduce toxicity was estimated by using a battery of bioassays with different types and durations of exposure, toxicity endpoints, and susceptibilities to specific modes of toxicant action. Bioindicator species representative of various ecosystems and comprising a wide range of taxa and exposure routes were applied. Analyses of the biotests results were carried out in relation to the residual concentrations of the tested xenobiotics, which were measured at different biodegradation times (chemical data presented in our earlier paper [19]).

#### 3.1.1. Producers

In the present work, the freshwater algae *R. subcapitata* and saltwater diatom *P. tricornutum*, species commonly used for ecotoxicological studies, were exposed to *U. isabellina* post-culture filtrates supplemented with 4-CP, 4-*t*-OP, and NP. As illustrated in Table 1, exposure of the microalgae species to the tested filtrates induced their growth inhibition during the assay, with the green alga showing a higher tolerance to the fungal post-culture samples than the diatom.

Similar differences in toxin sensitivity between marine and freshwater microorganisms were noted in studies on the effect of phenolic compounds on the growth and morphology of selected algae. Additionally, these studies showed that marine algae species were more sensitive to phenolic compounds than freshwater species, which was also shown in the results obtained in the present study. The diatom, compared to the green alga, was characterized by a smaller specific surface due to the small size of the cells. This led to faster absorption of phenolic xenobiotics in its cells and greater toxicity. Moreover, marine species have evolved to exist in highly stable environments, making them less resistant to stressors than freshwater species [21]. The present study showed that the EC_50_ values in the *R. subcapitata* assays performed for 24 h with *U. isabellina* post-culture filtrates treated with 4-CP, NP, and 4-*t*-OP increased by 6.4%, 1.8%, and 7.1%, respectively, compared to the values at the beginning of incubation, thus indicating the detoxification processes occurring during the microbiological degradation of the tested xenobiotics.

A reduction in ecotoxicity was also demonstrated in tests with *P. tricornutum*, where increases in EC_50_ values from 10.1% to 16.1% for 4-CP, from 6.5% to 19.6% for NP, and from 3.3% to 7.8% for 4-*t*-OP were observed for filtrates obtained from 0 and 24 h fungal cultures, respectively. Studies on toxicity changes during biological transformation and elimination of pollutants by assessing growth inhibition of various species of algae before and after treatment have also been reported in other works. A reduction in growth inhibition during enzymatic elimination of the antidepressant sertraline by treatment with the MnP-Tween 80 system was observed in tests with *R. subcapitata*. The use of the same species of algae as bioindicators showed ketoconazole detoxification after treatment with laccase from *Trametes versicolor* and a decrease in toxicity during biotransformation of tetracycline antibiotics by laccase treatment in the presence of the HBT redox mediator [22,23,24].

Plants are important components of ecosystems primarily because they are the main producers of food. Therefore, they are extremely helpful in monitoring pollutants [25,26]. Assays based on higher plants are characterized by low costs, quick test activation, and high sensitivity to a broad spectrum of contaminants; do not require qualified personnel; and offer a wide variety of toxicity endpoints [27,28,29]. In the present work, the phytotoxicity of the post-cultured filtrates was determined on the basis of the number of germinated seeds and root lengths of the tested plants. The results of contact tests with two species of dicotyledons and one species of monocotyledon used in the study showed different reactions of bioindicators to intermediates occurring in post-culture liquids (Table 2).

Numerous data indicate that dicotyledons are more sensitive to toxic substances than monocotyledons [30,31,32]. These findings are in agreement with the results obtained in this work for watercress and sorghum, which reveal that *L. sativum* is a more sensitive species for assessing changes in ecotoxicity during biodegradation processes than *S. saccharatum*. IR values ranged from 54.5% to 92.1% and from 21.0% to 65.8% for watercress and sorghum, respectively, whereas the GI values varied within the range from 11.7% to 45.9% (*L. sativum*) and from 34.2% to 79.0% (*S. saccharatum*) during the whole biodegradation process.

Generally, on the basis of the biotests with watercress and sorghum, after 24 h of incubation in all tested xenobiotic-fungus systems, a significant decrease in phytotoxicity was observed compared to the beginning of the experiments (Table 2). These results corresponded with the chemical data presented in our previous work [19], indicating a positive correlation between the amount of the analyzed xenobiotics during the *U. isabellina* cultures and the values of the tested endpoints in *L. sativum* and *S. saccharatum*. The observed reduction in the toxicity of post-culture filtrates proved the biotransformation of xenobiotics into intermediates with lower toxicity than the precursor compounds.

Considering the use of mustard in ecotoxicity tests, many studies point out that *S. alba* is a species with higher resistance to the presence of many organic and inorganic pollutants than other bioindicator plants [30,32]. However, unexpectedly, the obtained results showed that mustard was the most sensitive test plant to the contaminants present in the post-culture filtrates. Compared to the controls, for all fungal cultures supplemented with xenobiotics, 90% to 100% IG and 80% to 100% IR in *S. alba* tests were observed. In other studies assessing the phytotoxicity of soil from petrochemically contaminated sites, for several tested matrices, *S. alba* was found to be the most resistant species to soil chemicals belonging to the BTEX and PAH groups [33].

Our phytotoxicity data confirm that tolerance to stressors such as xenobiotics is a species-specific trait. The contaminants present in the fungal cultures underwent continuous biotransformation processes. These mechanisms significantly determined the bioavailability of the intermediates and thus their toxicity, which turned out to be the highest for mustard. Taking into account the obtained results, it seems interesting to carry out further analyses to determine the chemical profile of the filtrates to better understand the ecotoxicological response of *S. alba* to intermediates formed during 4-CP, NP, and 4-*t*-OP biodegradation. As a result, this species may become a key bioindicator of the toxicity of derivatives resulting from the degradation of phenolic xenobiotics.

#### 3.1.2. Consumers

In our previous studies, we applied two species of crustaceans, *D. magna* and *A. franciscana*, to evaluate changes in toxicity during microbial degradation of xenobiotics, and the mortality of the test organisms was used as the toxicity endpoint [19]. In the present work, we extended the study to a test that allows for rapid assessment of toxicity with the use of short-term exposure to *T. platyurus* larvae. This assay uses the inhibition of food ingestion (uptake of artificial particles) by the tested bioindicators as a result of exposure to toxic stress. Ingestion is considered to be an important indicator of toxicity, as this ecologically significant behavior directly affects growth and reproduction.

Our research showed that exposure of *T. platyurus* to post-culture filtrates resulted in inhibition of food ingestion particles by crustaceans in all tested systems, indicating the toxic nature of the contaminants present in the treated samples (Table 1). Nevertheless, based on the Rapidtoxkit test results, we found that after 24 h of incubation of fungal cultures supplemented with the tested xenobiotics, the toxicity of post-culture filtrates decreased 2.7-, 1.8-, and 1.7-fold for 4-CP, 4-*t*-OP, and NP, respectively. These results clearly show the effectiveness of biodegradation processes in reducing ecotoxicity from *U. isabellina* cultures, which was also observed in our previous studies for other species of bioindicators belonging to consumers [19]. Several previously published papers also demonstrated a decrease in sample toxicity for *T. platyurus* during biological and physicochemical methods of pollutant decomposition, e.g., active sludge treatment of wastewater originating from tank truck cleaning or photodegradation of aerucyclamide A [34,35].

#### 3.1.3. Decomposers

One of the most sensitive microbiological bioassays is based on the measurement of the bioluminescent activity of the marine bacteria *A. fischeri*. Data on the toxicity of post-culture filtrates for *A. fischeri* during the fungal elimination of 4-CP, 4-*t*-OP, and NP are shown in Table 1. Bioluminescence readings revealed a decrease in toxicity values during the biodegradation processes for all xenobiotics tested (27.9%, 23.6%, and 14.5% for 4-CP, 4-*t*-OP, and NP, respectively) at the end of the experiment. The rate of toxicity decline was correlated with the degree of xenobiotic biotransformation demonstrated in our earlier paper [19]. These results suggested that fungal degradation led to metabolites that were less toxic than the precursor compounds. It is considered that along with the enhancement of the lipophilic character of compounds, their ecotoxicity increases, which strongly inhibits cellular respiration and disturbs bioluminescence [17,36]. Considering the structure of the intermediates, we identified earlier. It is suggested that the processes of NP, 4-*t*-OP, and 4-CP biotransformation by the fungus (aliphatic chain reduction, hydroxylation) led to the formation of more polar derivatives exhibiting reduced toxicity. More hydrophilic and less toxic metabolites than the precursor compounds in the *A. fischeri* test were also noted during the degradation of NP by the fungus *Thielavia* sp HJ22 and polychlorinated biphenyls (PCBs) by *Pleurotus ostreatus* and *Irpex lacteus* strains [17,37].

**Figure 1:**
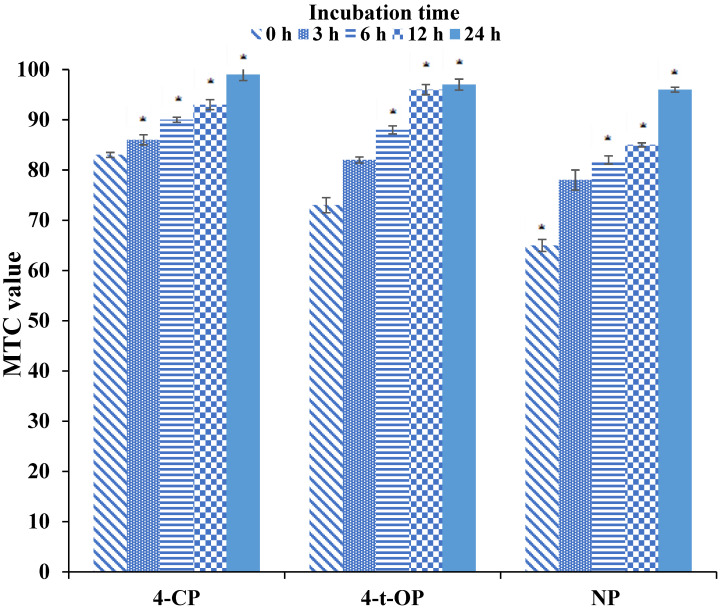
Mean MTC values in the MARA test with 4-CP, NP, and 4-*t*-OP fungal culture exposure. An asterisk (*) indicates statistically significant differences from the control (*p* ≤ 0.05).

The toxicity of by-products formed during the biodegradation of the tested xenobiotics by *U. isabellina* was also assessed using the multispecies MARA test. The species used in this assay show different sensitivities to toxicants, and the results obtained give a unique pattern of the microbial growth response (toxic fingerprint) to the tested samples. The data received from the MARA test are summarized in Figure 1. Additionally, MTC values for each strain are represented in Figure S2.

The dose-response relationship of the different MARA species exposed to the post-culture filtrates was different for each of the xenobiotics tested. Such findings are typical of the MARA bioassay, as they reflect the wide genetic diversity of the species and thus significant differences in susceptibility among microorganisms. Exposure to fungus-treated xenobiotic-polluted media resulted in the growth reduction in almost all tested strains compared to negative controls.

The most sensitive microorganism to the cultures supplemented with NP and 4-*t*-OP was *B. diminuta*, while the strongest effect of 4-CP-treated cultures on growth inhibition was observed for *C. testosteroni*. *S. rubidaea* and *C. freundii* were also susceptible to the tested filtrates. The obtained data correspond with previously presented studies showing that these strains were characterized by high sensitivity among MARA species to the presence of other toxicants, e.g., naproxen for *B. diminuta* or doxycycline for *S. rubidaea* and *C. freundii* [38,39]. Relatively low MTC values and, consequently, higher adverse effects of the tested pollutants on the activity of microorganisms commonly found in water and soil ecosystems indicate that these species may be useful markers of treatment efficiency environments contaminated with both phenolic xenobiotics and their derivatives. The results of the MARA assay showed that *P. aurantiaca* was the most tolerant microorganism to the toxic effect of post-culture filtrates. Similar findings were noted during the exposure of the strain to naproxen. Relatively high resistance to the tested matrices may result from the fast adaptation mechanisms of *P. aurantiaca* to toxins present in the environment (e.g., by stabilization of membrane structures) and may also be related to the production of enzymes involved in the degradation of toxic products [38].

The results indicate that the average MTC values obtained for the post-cultured filtrates increase with increasing incubation time of *U. isabellina* with the tested toxicants. This finding indicates, similar to other biotests that the intermediates formed during biotransformation are less toxic than the precursor compounds. Our results confirm that the comparative sensitivity of the various bioindicators for the matrices tested cannot be generalized but should be assessed on a case-by-case basis.

#### 3.1.4. Yeast Screen Assay—Endocrine Activity

Because many estrogenic properties have been reported in the literature, they receive a large amount of attention, and relatively little is known about the influence of various xenobiotics on androgen receptors; in the present work, both the estrogenic and androgenic properties, as well as the anti-estrogenic and anti-androgenic properties of 4-CP, 4-*t*-OP, and NP, were investigated. Moreover, the endocrine activity of post-culture liquids obtained after incubation with *U. isabellina* with the tested alkylphenols was also determined. The endocrine properties of the tested compounds are summarized in Table 3.

Our studies have confirmed that NP is a substance with estrogenic properties, which was also presented in other publications [40,41,42]. In the present study, estrogenic properties were also reported for cumylphenol (Figure S3). However, this activity was significantly lower than that of the standard oestradiol (E2). The estrogenic activity of the tested alkylphenols turned out to be 1000- and 10,000-times lower than the activity of the standard for NP and 4-CP, respectively. On the basis of the obtained results, it can also be stated that none of the tested substances show androgenic activity (Table 3). No changes in β-galactosidase activity in the presence of NP or bisphenol A (BPA) were noted by Park et al. [40], which is in agreement with the presented data. Literature data suggest that compounds that exhibit estrogenic properties may also show anti-androgenic activity [43]. Examples of such compounds include BPA and 4-*t*-OP [44]. Therefore, in the next stage, the anti-androgenic properties of selected phenols were also assessed.

The conducted analyses showed that 4-*t*-OP and 4-CP exhibit anti-androgenic properties and that the addition of these substances reduces the activity of DHT by approximately 30–40%, depending on the analyzed sample. The literature data indicate that alkylphenols such as BPA, NP, or OP can interact with androgen receptors. These properties have not yet been demonstrated for 4-CP [45]. To our knowledge, this is the first report to demonstrate the anti-androgenic activity of cumylphenol. On the basis of the obtained results, it can also be concluded that NP does not show anti-androgenic activity. On the other hand, Lee et al. [46] indicated that NP is an active anti-androgen.

In the next stage of the work, the post-culture fluids after incubation with *U. isabellina* with selected phenols were evaluated for endocrine properties. The conducted analyses showed that none of the tested post-culture fluids obtained after incubation of *U. isabellina* with NP or 4-CP showed estrogenic activity, regardless of the incubation time and concentration of the tested sample. In the test determining the anti-androgenic activity of the tested post-culture liquids, it was shown that properties depend on the time of incubation of *U. isabellina* with the substrates (Table S4). The highest decrease in DHT activity was noted in trials from 0 h. Along with the extension of the incubation time, the anti-androgenic activity of the tested fluids decreased, which indicates that the biodegradation process of the discussed alkylphenols leads to their detoxification.

### 3.2. Growth Ability of U. isabellina in Leachate Presence

**Figure 2:**
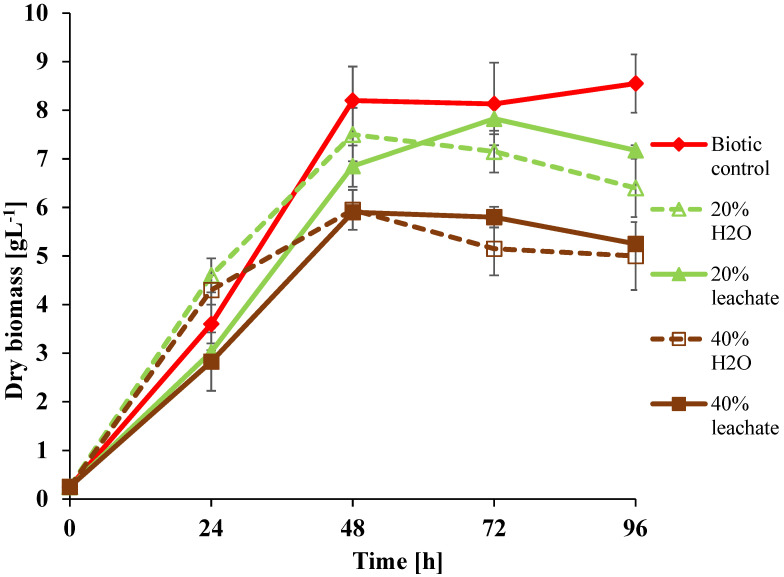
Effect of supplementation with landfill leachate (20% and 40% of the volume) on the growth of *U. isabellina* during 96 h of incubation.

The growth of *U. isabellina* on Sabouraud medium containing 20% and 40% landfill leachate originating from the closed dangerous waste landfill of the former “Boruta” Dye Factory in Zgierz, Poland, is presented in Figure 2.

The obtained results show that in 24 h of incubation, the amount of biomass in the culture with 20% of the landfill leachate was similar to that in the controls with 20 and 40% water (instead of landfill leachate), as well as in the biotic control without any supplements. In the set with 40% landfill leachate, a significantly lower amount of biomass was observed in relation to that in the adequate control supplemented with water. In the following hours of incubation, the difference within both cultures decreased, and at the end of the experiment, the difference was at the same level but much lower than that in the biotic control without any supplements. The obtained results suggest that the fungus is able to adapt to the unfavorable conditions caused by the landfill leachate components, even in the case of additional landfill leachate introduction.

The basic analyses of the landfill leachate (Table S1) revealed a high worth of COD (chemical oxygen demand) as well as total organic carbon (TOC) content and relatively low biochemical oxygen demand (BOD), which suggests the presence of toxic organic and inorganic (high value of conductivity) components for the leachate microbiota. The investigated leachate originates from a dye industry waste landfill located within the boundary of the city Zgierz in the central region of Poland (Figure 1). The landfill is currently closed but was in use from 1995 to 2015 predominantly by the former “Boruta” Dye Industry Plant in Zgierz, Poland. The post-production waste of the former Boruta factory was stored in iron containers, barrels, or bins and then covered with a layer of sand, ash, and municipal waste as well as a layer of ash, gypsum, and asbestos [47,48].

The detailed chemical analyses of the leachate (Table S2) showed additionally high amounts of iron (the most likely the result of metal container corrosion) and VPs. VPs are benzene hydroxyl derivatives and other aromatic hydroxyl chemicals that distill water vapor from an acid solution and, under specified standard conditions, give a color reaction with 4-aminoantipyrine (PN-EN ISO 14402:2004). VPs are synthesized by microorganisms and are formed during the biotransformation and biodegradation processes of some natural compounds and xenobiotics, e.g., non-volatile phenols, aromatic amino acids, polycyclic aromatic hydrocarbons (PAHs), and polyphenols [49]. The leachate from the landfill was sent by the industrial liquid waste system of the former “Boruta” factory to the municipal and industrial wastewater plant in Zgierz, Poland. The possible ability of the tested fungus to degrade and detoxify VPs present in the leachate could be useful for the wastewater plant in Zgierz.

### 3.3. VP Utilization by U. isabellina

**Figure 3:**
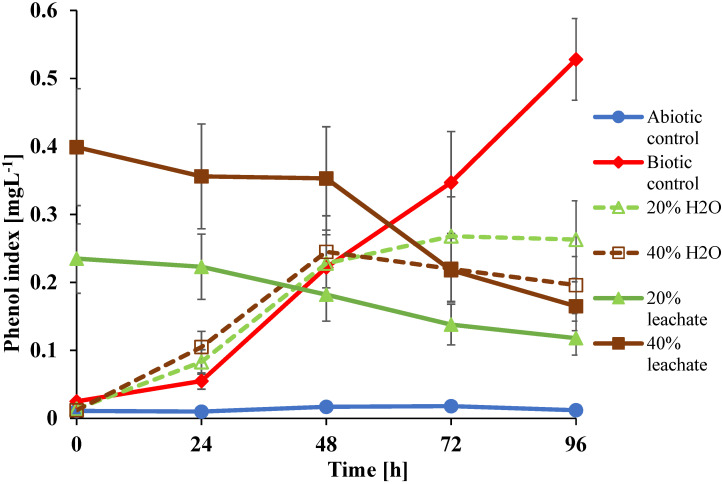
Volatile phenol content during *U. isabellina* cultures with the addition of 20% and 40% landfill leachate.

Analyses of VPs in cultures of *U. isabellina* (Figure 3) revealed that in the culture without any supplements (biotic control), the fungus released VPs into the medium over the course of the experiment until the end of incubation at 96 h.

Fungi are able to produce and use numerous volatile organic compounds (VOCs), including VPs [50,51]. In the investigated control culture with 20% water (instead of landfill leachate) for 48 h, a decrease in VP formation was noticed.

In the case of the control set with 40% water (in place of landfill leachate) after 48 h, even a decrease in previously released VPs was observed. These phenomena are correlated with a very rapid breakdown of the VP content after 48 h of culturing in the set with 40% landfill leachate and indicate that VPs are used by *U. isabellina*, especially in the stationary phase growth phase when the easily and quickly metabolized components of the growth medium are consumed. This conclusion also seems to be confirmed by a slight decrease in the VPs content in the first 24 h of the experiment in the culture supplemented with 20% leachate and gradual acceleration of this process in the further hours of incubation.

### 3.4. Evaluation of Sample Toxicity from U. isabellina Cultures Supplemented with Leachate

To estimate the detoxification capability of the tested fungus in relation to the post-industrial landfill leachate, toxicity tests using organisms representing different levels of the trophic chain (consumer, decomposer, producer) were carried out. In many cases, biotests containing bioindicators belonging to different taxonomic groups and exhibiting various key functions in the ecosystem have been applied to provide a comprehensive assessment of raw and treated landfill leachate ecotoxicity [28].

In all of our tests, a significant correlation between the toxicity reduction in filtrates from *U. isabellina* culture in the presence of different leachate concentrations and the decrease in VPs in these samples was observed. Reductions in toxicity: 3.8-, 4.9-, and 1.9-fold for the 20% leachate volume and 3.7-, 4.6-, and 2.1-fold for the 40% leachate volume in fungal cultures were achieved during the 96 h of biodegradation experiments for *A. franciscana*, *D. magna,* and *A. fischeri*, respectively (Table 4).

Other studies using *A. franciscana* as a bioindicator showed a 21% decrease in the harmful potential of landfill leachate during treatment processes, which was a similar value to our results [52]. A significant reduction in toxicity for 60% of the landfill leachate as a result of biological treatment over a 35-day period was also observed in the *D. magna* tests [53].

The effectiveness of decreasing toxicity after biological treatment of landfill leachate has also been successfully demonstrated in many studies by measuring the bioluminescent activity of bacteria. The application of *A. fischeri* in studies carried out by Kalka (2012) [54] revealed a reduction in toxicity by 67.2% in the treated leachate, while Kalčíková et al. [55], using the same test, showed a reduction of 33.4% in harmfulness in leachate during the growth of the fungus *Dichomitus squalens* after 3 days of incubation.

Therefore, reports indicate that toxicity assessment with *A. franciscana*, *D. magna,* and *A. fischeri* is convenient, effective, and useful for monitoring the effectiveness of biological leachate treatment processes [56].

In the present work, toxicity results varied depending on the test organism and selected endpoints, with *D. magna* identified as the most sensitive organism. The lower resistance of daphnids and other freshwater crustaceans to the toxic effect of leachate compared to other bioindicators has already been demonstrated [57,58]. The high toxicity of the tested landfill leachate to *D. magna* may be due to the high conductivity and the presence of heavy metals, especially the high TOC values that exceed the standard by more than 30 times (Table S1). The high content of organic matter may reduce the oxygen content in the sample, negatively affecting the survival rate of daphnids [59].

The application of luminescent bacteria in the present study revealed the toxic nature of the landfill leachate; however, among the tested bioindicators, *A. fischeri* showed the lowest sensitivity. A lower frequency of toxic reactions in response to landfill leachate in *A. fischeri* compared to test organisms representing different trophic levels was also reported in another study [57]. It has been suggested that the reason for such findings may be the presence of substances with insecticidal and herbicidal properties in the leachate. The harmful impact of leachate on the luminescent activity of *A. fischeri* could have resulted from the coexistence of several metals, especially Fe^2+^, the amount of which significantly exceeded the norms. It was noted that the inhibition of bioluminescence by the leachate was positively correlated with the metals present, including Fe^2+^, that could be adsorbed and trapped in the exo-polysaccharide layer of the bacteria [60].

Our findings suggest that the toxicity decrease in filtrates in the test with *A. fischeri* may be caused not only by the biodegradation processes of organic substances but also by heavy metal ion biosorption or/and bioaccumulation mechanisms. This is confirmed by the results of our earlier studies, in which we demonstrated the ability of *U. isabellina* to remove selected heavy metals from the growth medium [14].

All filtrate samples were evaluated as phytotoxic based on *S. saccharatum* seed germination and root growth, especially at higher tested leachate concentrations. It was found that the use of 20% and 40% of the leachate in fungal cultures resulted in root growth inhibition of 32.5% and 59.1%, respectively, for the filtrates from the start of cultivation. A correlation between a higher leachate content and a greater percent of root growth inhibition of exposed plants was also demonstrated by Šourková et al. [61], who noticed a similar degree of *S. alba* root growth reduction using 25% and 50% leachate. These findings indicate that the use of large amounts of leachate disrupts the defense system and the metabolism of bioindicator plant species. The phytotoxicity test also showed that toxicity declined in the filtrates obtained during *U. isabellina* incubation with the addition of leachates (Table S5). At 96 h of cultivation, the GI increased by 18.8% and 19.1% for post-culture samples containing 20% and 40% leachate, respectively.

Studies on root growth inhibition in the presence of tested supernatants also revealed a reduction in their harmful effects on *S. saccharatum*. The growth inhibition of the roots treated with the 4-day post-culture filtrates was reduced by approximately 19% compared to the starter cultures for both the 20% and 40% leachate volumes. The toxic effect of leachate on *S. saccharatum* root elongation may be caused by the high conductivity value. A strong dependence of *S. saccharatum* root growth inhibition on conductivity was also demonstrated in studies on the phytotoxicity assessment of leachate from a controlled municipal landfill in Gdansk (Poland) [57]. The availability value of water-soluble pollutants is high for plants; therefore, they may have the strongest impact on bioindicators, which present the first level of the trophic chain. The results of contact tests with plant seeds also showed an increase in toxicity during the first 48 h of biodegradation processes. This suggests that VPs and other organic contaminants present in the tested leachate could have been degraded into by-products characterized by higher toxicity than the precursor compounds. In conclusion, the obtained data clearly indicate that the microbiological processes of compound metabolism contained in leachate, including VPs, result in their detoxification.

The results obtained in the presented study indicate for the first time a non-lignolytic fungus capable of decomposing and detoxifying phenolic compounds that disrupt the functioning of the endocrine system, both in laboratory conditions and from highly contaminated post-industrial environments.

## 4. Conclusions

The results obtained in the study provide accurate information on the reduction in cumulative harmful effects of compounds mixtures formed during the degradation of 4-CP, NP, and 4-*t*-OP by *U. isabellina* strain and decrease their adverse effect on biota. Moreover, the data presented in this work show that the fungal strain also has the ability to eliminate and detoxify VPs from leachate produced by landfills post-industrial waste. The use of a battery of biotests in our study provided information on the biological activity of leachate treated with biological treatment processes. These studies clearly demonstrate the importance of a multispecies approach not only for determining leachate toxicity but also for gaining valuable information on the efficiency of microbial bioremediation processes, which is the basis for the development of additional treatment strategies for contaminated aquatic environments. Thus, it can be concluded that the non-ligninolytic fungus *U. isabellina* shows potential usefulness in bioremediation processes not only in environments contaminated with specific phenolic xenobiotics but also in industrial leachates with similar chemical profiles.

The results of the biotests presented in the paper demonstrate sufficient evidence to estimate the effectiveness of hazard reduction by biodegradation processes, indicating the potential of the tested fungal strain to be used as an attractive tool for bioremediation of areas contaminated with phenolic xenobiotics. This creates the prospect of the possible inclusion of this fungus in wastewater treatment programs as a promising alternative to the often costly and not eco-friendly physicochemical methods of reducing toxic pollutants.

## Figures and Tables

**Table 1:** Assessment of the acute toxicity effects of *U. isabellina* post-culture filtrates with the addition of 4-CP, NP, or 4-*t*-OP by using different bioindicator species.

Xenobiotic	Time (h)	*P. tricornutum*	*R. subcapitata*	*T. platyurus*	*A. fischeri*
		EC50			
4-CP	0	10.1 ± 0.3	22.2 ± 0.5	3.6 ± 0.3	46.2 ± 1.6
	3	9.5 ± 0.5	19.7 ± 0.4	4.0 ± 0.5	59.0 ± 1.8
	6	10.7 ± 0.2	20.1 ± 0.4	5.0 ± 0.4	63.1 ± 2.1
	12	10.9 ± 0.7	19.8 ± 0.3	7.6 ± 0.5	70.4 ± 1.6
	24	16.1 ± 0.7	28.6 ± 0.5	9.8 ± 0.4	74.1 ± 1.5
4-*t*-OP	0	3.3 ± 0.3	4.4 ± 0.2	10.1 ± 0.7	52.6 ± 2.0
	3	4.2 ± 0.5	4.3 ± 0.3	13.2 ± 0.3	62.1 ± 1.1
	6	5.1 ± 0.3	4.5 ± 0.2	14.3 ± 0.6	71.1 ± 1.7
	12	3.8 ± 0.4	5.8 ± 0.3	14.7 ± 0.3	75.7 ± 2.0
	24	7.8 ± 0.5	6.2 ± 0.1	17.9 ± 0.7	74.2 ± 1.4
NP	0	6.5 ± 0.7	4.9 ± 0.2	4.5 ± 0.4	58.3 ± 1.5
	3	6.3 ± 0.3	5.1 ± 0.3	5.9 ± 0.4	70.8 ± 1.3
	6	6.0 ± 0.6	6.4 ± 0.3	5.6 ± 0.2	74.2 ± 1.5
	12	7.4 ± 0.4	9.1 ± 0.2	5.4 ± 0.2	65.2 ± 1.7
	24	19.6 ± 0.7	12 ± 0.4	7.9 ± 0.5	72.8 ± 2.1

**Table 2:** Changes in the phytotoxicity of post-culture filtrates before and after xenobiotic biodegradation processes.

Xenobiotic	Incubation Time (h)	Germination Inhibition (PE%)			Root Growth Inhibition (PE%)			Germination Index (%)		
		Sa ^1^	Ss ^2^	Ls ^3^	Sa ^1^	Ss ^2^	Ls ^3^	Sa ^1^	Ss ^2^	Ls ^3^
NP	0	100 ± 0	0 ± 0	0 ± 0	100 ± 0	59.5 ± 4.0	72.5 ± 3.0	0 ± 0	40.5 ± 1.8	27.5 ± 2.2
	3	80 ± 5	0 ± 0	0 ± 0	78.9 ± 3.5	42.8 ± 2.6	67.1 ± 2.9	4.2 ± 1.1	57.2 ± 2.5	32.9 ± 1.9
	6	90 ± 3	10 ± 3	0 ± 0	88.6 ± 2.8	25.3 ± 2.7	60.3 ± 3.3	1.4 ± 0.2	67.0 ± 3.0	39.8 ± 2.5
	12	100 ± 0	0 ± 0	0 ± 0	100 ± 0	25.5 ± 4.5	54.5 ± 2.0	0 ± 0	74.7 ± 3.6	45.6 ± 2.8
	24	90 ± 3	0 ± 0	0 ± 0	90.1 ± 3.1	21.0 ± 3.5	54.1 ± 3.5	1.0 ± 0.2	79.0 ± 2.8	45.9 ± 2.2
4-*t*-OP	0	100 ± 0	0 ± 0	0 ± 0	100 ± 0	65.3 ± 3.1	92.1 ± 3.5	0 ± 0	34.8 ± 1.7	11.7 ± 1.0
	3	90 ± 5	0 ± 0	0 ± 0	99.2 ± 1.0	65.8 ± 2.5	87.1 ± 4.1	0.07 ± 0.1	34.2 ± 1.1	12.9 ± 1.7
	6	100 ± 0	0 ± 0	0 ± 0	100 ± 0	59.9 ± 2.6	87.6 ± 2.6	0 ± 0	40.1 ± 1.0	12.4 ± 1.5
	12	90 ± 5	0 ± 0	0 ± 0	97.7 ± 1.0	56.0 ± 2.6	83.2 ± 3.0	0.23 ± 0.1	44.0 ± 1.5	16.7 ± 1.8
	24	100 ± 0	0 ± 0	0 ± 0	100 ± 0	51.1 ± 2.0	81.4 ± 2.2	0 ± 0	48.9 ± 1.8	18.5 ± 1.5
4-CP	0	90 ± 3	0 ± 0	0 ± 0	98.9 ± 1.2	59.9 ± 4.2	84.4 ± 4.1	0.22 ± 0.1	40.6 ± 3.6	18.6 ± 2.2
	3	90 ± 3	0 ± 0	10 ± 3	95.8 ± 2.7	55.5 ± 3.0	81.1 ± 3.6	0.42 ± 0.1	44.5 ± 2.1	18.9 ± 3.0
	6	90 ± 0	0 ± 0	0 ± 0	95.2 ± 3.8	55.5 ± 3.5	77.3 ± 3.0	0.48 ± 0.1	44.5 ± 2.1	22.8 ± 2.5
	12	100 ± 0	0 ± 0	0 ± 0	100 ± 0	48.5 ± 4.0	76.9 ± 3.3	0 ± 0	51.5 ± 3.8	23.1 ± 2.6
	24	90 ± 3	0 ± 0	0 ± 0	95.3 ± 3.4	36.5 ± 3.2	63.9 ± 3.6	0.45 ± 0.1	63.5 ± 4.2	36.0 ± 3.1

^1^ Sa—*S. alba*; ^2^ Ss—*S. saccharatum*; ^3^ Ls—*L. sativum*.

**Table 3:** Activity of test xenobiotics in the XenoScreen YES/YAS Endocrine Disruptor Assay ^1^.

Xenobiotic	YES	YAS	Anti-YES	Anti-YAS
4-CP (concentration range: 5000–15 µg L^−1^)	Estrogenic activity (about 10.000 times lower than estradiol)	Lack of androgenic properties	Lack of anti-estrogenic properties	Weak anti-androgenic activity
4-*t*-OP (concentration range: 5000–15 µg L^−1^)	Lack of estrogenic activity	Lack of androgenic properties	Lack of anti-estrogenic properties	Weak anti-androgenic activity
**NP** (concentration range: 200,000–1500 µg L^−1^)	Estrogenic activity (about 1.000 times lower than estradiol)	Lack of androgenic properties	Lack of anti-estrogenic properties	Lack of anti-androgenic properties

^1^ For each system, the activity of estradiol, DHT, flutamide, 4-hydroxy tamoxifen was determined, and also the effect of suppressing the action of individual compounds was checked.

**Table 4:** Ecotoxicity data (EC50 and LC50 values) for *D. magna*, *A. franciscana,* and *A. fischeri* assays in the presence of post-culture filtrates treated with the landfill leachate.

Landfill Leachate (%)	Time (h)	*A. fischeri*	*A. fransciscana*	*D. magna*
20	0	34.2 ± 0.6	13.4 ± 0.6	9.1 ± 0.6
	24	40.1 ± 0.8	15.6 ± 0.9	14.5 ± 1.1
	48	45.1 ± 1.0	19.7 ± 1.1	26.9 ± 1.4
	72	56.4 ± 0.9	25.6 ± 0.5	30.4 ± 1.0
	96	66.9 ± 1.3	50.4 ± 1.2	44.1 ± 1.5
40	0	15.9 ± 0.4	7.8 ± 1.0	4.4 ± 0.2
	24	24.7 ± 0.5	10.1 ± 0.9	7.2 ± 0.9
	48	28.2 ± 0.9	15.2 ± 1.0	10.0 ± 0.5
	72	31.1 ± 0.5	17.4 ± 1.0	11.5 ± 0.3
	96	34.0 ± 1.0	28.6 ± 1.5	20.2 ± 1.2

## Data Availability

Data are contained within the article.

## Supplementary Materials

The following supporting information can be downloaded at: https://www.mdpi.com/article/10.3390/ijerph19074093/s1, Table S1: Basic analysis of the landfill leachate collected from the hazardous waste landfill of the former “Boruta” dye production plants in Zgierz; Table S2: Chemical contaminants of the landfill leachate; Table S3: Toxicity bioassays selected for the ecotoxicological analysis of filtrates from *U. isabellina* cultures supplemented with xenobiotics and landfill leachate; Table S4: Anti-androgenic activity of post-culture filtrates obtained after incubation of *U. isabellina* with NP or 4-CP (post-culture liquid concentration −4%); Table S5: Plant biological endpoints for testing the phytotoxicity of fungal post-culture filtrates supplemented with landfill leachate; Figure S1: Location of leachate collection from post-industrial landfills. Study area location in Zgierz city (Poland, Central Europe). A. The municipal and industrial wastewater treatment plant, B. The former “Boruta” Dye Industry Plant area, C. The closed landfill for hazardous waste of the former “Boruta” Dye Industry Plant, D. The closed energy ash and gypsum landfill; Figure S2: MARA species responses (MTC value) to *U. isabellina* cultures treated with test xenobiotics: a—4-CP, b—NP, c—4-*t*-OP; Figure S3: Estrogenic activity of 4-CP, 4-*t*-OP and NP.
