# Supplementary material for: Ecotoxicological Estimation of 4-Cumylphenol, 4-t-Octylphenol, Nonylphenol, and Volatile Leachate Phenol Degradation by the Microscopic Fungus Umbelopsis isabellina Using a Battery of Biotests
Source: Int J Environ Res Public Health. 2022 Mar 30;19(7):4093. doi: 10.3390/ijerph19074093 (PMC8998573; doi:10.3390/ijerph19074093)
Supplement: Supplementary file 1 [file ijerph-19-04093-s001.zip › ijerph-1620969-Supplementary materials.pdf]

International Journal of
Environmental Research and Public Health

Supplementary Materials

Ecotoxicological estimation of 4-cumylphenol, 4-*t*-octylphenol, nonylphenol and volatile leachate phenol degradation by the microscopic fungus *Umbelopsis isabellina* using a battery of biotests

Tomasz Janicki [1], Andrzej Długoński [2,3], Aleksandra Felczak [1], Jerzy Długoński [1] and Mariusz Krupiński [1,*]

[1] Department of Industrial Microbiology and Biotechnology, Faculty of Biology and Environmental Protection, University of Lodz, Banacha 12/16 Street, 90-237 Lodz, Poland; janickithomas@gmail.com (T.J.); aleksandra.felczak@biol.uni.lodz.pl (A.F.); jerzy.dlugonski@biol.uni.lodz.pl (J.D.); mariusz.krupinski@biol.uni.lodz.pl (M.K.)
[2] Institute of Biological Sciences, Faculty of Biology and Environmental Sciences, Cardinal Stefan Wyszyński University in Warsaw, 1/3 Wóycickiego Street, 01-038 Warsaw, Poland; andrzej.dlugonski@biol.uni.lodz.pl (A.D.)
[3] Institute of Ecology and Environmental Protection, Faculty of Biology and Environmental Protection, University of Lodz, Banacha 12/16 Street, 90-237 Lodz, Poland; andrzej.dlugonski@biol.uni.lodz.pl (A.D.)
* Correspondence: mariusz.krupinski@biol.uni.lodz.pl

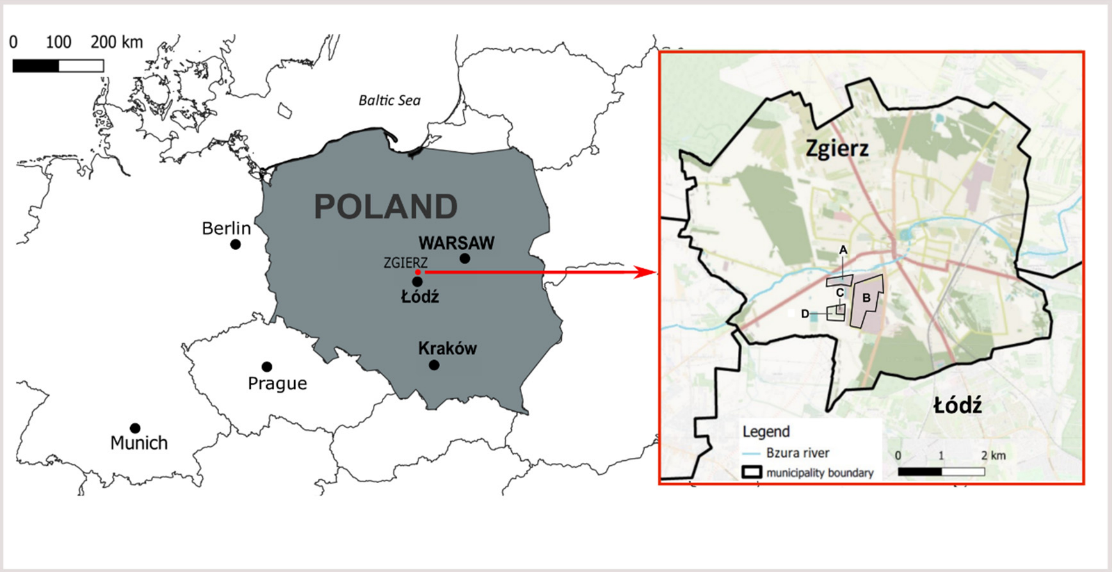

Figure S1. Location of leachate collection from post-industrial landfills. Study area location in Zgierz city (Poland, Central Europe). A. The municipal and industrial wastewater treatment plant, B. The former "Boruta" Dye Industry Plant area, C. The closed landfill for hazardous waste of the former "Boruta" Dye Industry Plant, D. The closed energy ash and gypsum landfill.

Table S1. Basic analysis of the landfill leachate collected from the hazardous waste landfill of the former "Boruta" dye production plants in Zgierz.

Parameter	Method	Unit	Value	Standard
pH	PN-EN ISO 10523:12	pH	7.2 ± 0.4	6.5 – 9.0
Colour	PN-EN ISO 7887:2012	Pt-Co	2920 ± 230	-
Unit weight	ASTM D854	g cm^{-3}	1.018 ± 0.003	-
Conductivity	PN-EN 27888:1999	µS	11805 ± 673	-
COD_{Mn}	PN-ISO 15705:2005	mg L^{-1} O_2	348.8 ± 92.4	125
TOC	PN-EN 1484:1999	mg L^{-1} C	1040.5 ± 190.4	30
BOD_5	PN-EN 1899-2:2002	mg L^{-1} O_2	300 ± 15.2	-

Table S2. Chemical contaminants of the landfill leachate.

Parameter	Method	Unit	Value	Standard
Nitrites	PN-EN ISO 13395:2001	mg L^{-1} NO_2	0.026 ± 006	1
Nitrates	PN-EN ISO 10304-1:2009	mg L^{-1} NO_3	< 1.7	30
Sulphates	PN-EN ISO 10304-1:2009	mg L^{-1} SO_4	15 ± 3	500
Chlorides	PN-EN ISO 10304-1:2009	mg L^{-1} Cl	2.1 ± 0.4	1000
Cyanides (free)	PN-EN ISO 14403-2:2012	mg L^{-1}	< 0.008	-
Cyanides (bound)	PN-EN ISO 14403-2:2012	mg L^{-1}	0.045 ± 0.008	-
Antimony	PN-EN ISO 11885:2009	mg L^{-1} Sb	<0.020	0.3
Arsenic	PN-EN ISO 11885:2009	mg L^{-1} As	<0.020	0.1
Barium	PN-EN ISO 11885:2009	mg L^{-1} Ba	0.37 ± 0.08	2
Beryllium	PN-EN ISO 11885:2009	mg L^{-1} Be	<0.004	1
Boron	PN-EN ISO 11885:2009	mg L^{-1} B	20.35 ± 4.68	1
Chromium (total)	PN-EN ISO 11885:2009	mg L^{-1} Cr_{total}	0.055 ± 0.011	0.1
Zinc	PN-EN ISO 11885:2009	mg L^{-1} Zn	0.067 ± 0.015	2
Aluminum	PN-EN ISO 11885:2009	mg L^{-1} Al	0.071 ± 0.016	3
Cadmium	PN-EN ISO 11885:2009	mg L^{-1} Cd	0.0015 ± 0.0003	0.4

Cobalt	PN-EN ISO 11885:2009	mg L^{-1} Co	<0.002	1
Manganese	PN-EN ISO 11885:2009	mg L^{-1} Mn	0.17 ± 0.04	-
Copper	PN-EN ISO 11885:2009	mg L^{-1} Cu	0.014 ± 0.003	0.5
Molybdenum	PN-EN ISO 11885:2009	mg L^{-1} Mo	0.057 ± 0.009	1
Nickel	PN-EN ISO 11885:2009	mg L^{-1} Ni	0.23 ± 0.04	0.5
Lead	PN-EN ISO 11885:2009	mg L^{-1} Pb	0.023 ± 0.004	0.5
Selenium	PN-EN ISO 11885:2009	mg L^{-1} Se	<0.050	-
Silver	PN-EN ISO 11885:2009	mg/L Ag	<0.010	0.1
Thallium	PN-EN ISO 11885:2009	mg L^{-1} Tl	<0.020	1
Titanium	PN-EN ISO 11885:2009	mg L^{-1} Ti	0.024 ± 0.005	1
Vanadium	PN-EN ISO 11885:2009	mg L^{-1} V	<0.006	2
Iron	PN-EN ISO 11885:2009	mg L^{-1} Fe	30.88 ± 5.87	10
Mercury	EPA 7473 02.2007	mg L^{-1} Hg	0.0014 ± 0.0002	0.06
Volatile phenols	PN-EN ISO 14402 : 2004	mg L^{-1}	1.68 ± 0.48	0.1
Petroleum hydrocarbons	PN-EN ISO 9377-2:2003	mg L^{-1}	7.2 ± 2.3	15

Table S3. Toxicity bioassays selected for the ecotoxicological analysis of filtrates from *U. isabellina* cultures supplemented with xenobiotics and landfill leachate.

**Trophic level**	**Species**	**Measure of toxic effect (toxicological endpoints)**	**4NP, 4-CP, 4-*t*-OP samples analyzed**	**VPs samples analyzed**
Decomposer	*Aliivibrio fischeri*	Bioluminescence activity	X	X
	Microbacterium sp.	Growth rate inhibition	X	
	Brevundimonas diminuta	Growth rate inhibition	X	
	Citrobacter freudii	Growth rate inhibition	X	
	Comamonas testosteroni	Growth rate inhibition	X	
	Enterococcus casseliflavus	Growth rate inhibition	X	

	Delftia acidovorans	Growth rate inhibition	X	
	Kurthia gibsonii	Growth rate inhibition	X	
	Staphylococcus warneri	Growth rate inhibition	X	
	Pseudomonas aurantiaca	Growth rate inhibition	X	
	Serratia rubidaea	Growth rate inhibition	X	
	Pichia anomala	Growth rate inhibition	X	
	Saccharomyces cerevisiae	Estrogenic/anti-estrogenic activity<br>Androgenic/anti-androgenic activity	X<br>X	
Producer	*Sorghum saccharatum*	Seed germination rate, root length reduction	X	X
	Lepidium sativum	Seed germination rate, root length reduction	X	
	Sinapis alba	Seed germination rate, root length reduction	X	
	Rhapidocelis subcapitata	Growth rate inhibition	X	
	Phaeodactylum tricornutum	Growth rate inhibition	X	
Consumer	*Thamnocephalus platyurus*	Reduction or complete cessation of food intake	X	
	Daphnia magna	Mortality	X[a]	X
	Artemia franciscana	Mortality	X[a]	X

[a] – tests were carried out in previous studies [19]

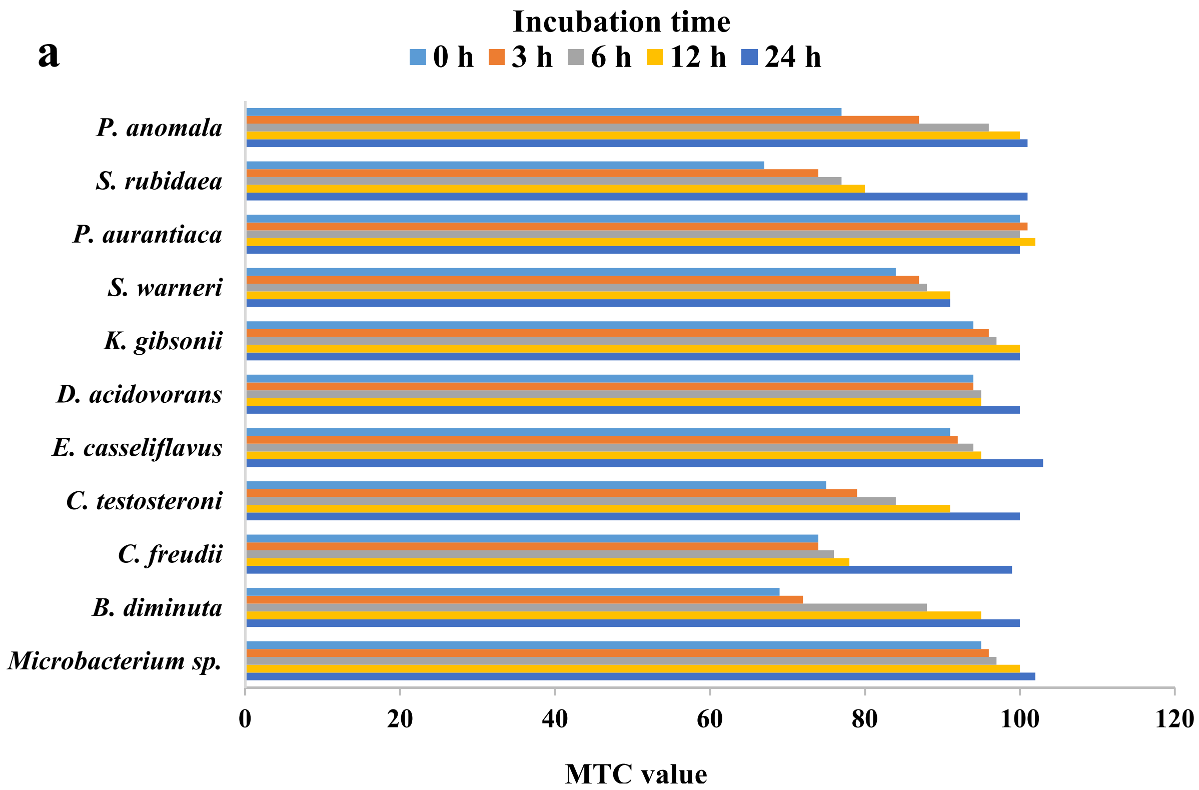
a
Incubation time
0 h
3 h
6 h
12 h
24 h
P. anomala
S. rubidaea
P. aurantiaca
S. warneri
K. gibsonii
D. acidovorans
E. casseliflavus
C. testosteroni
C. freudii
B. diminuta
Microbacterium sp.
0
20
40
60
80
100
120
MTC value

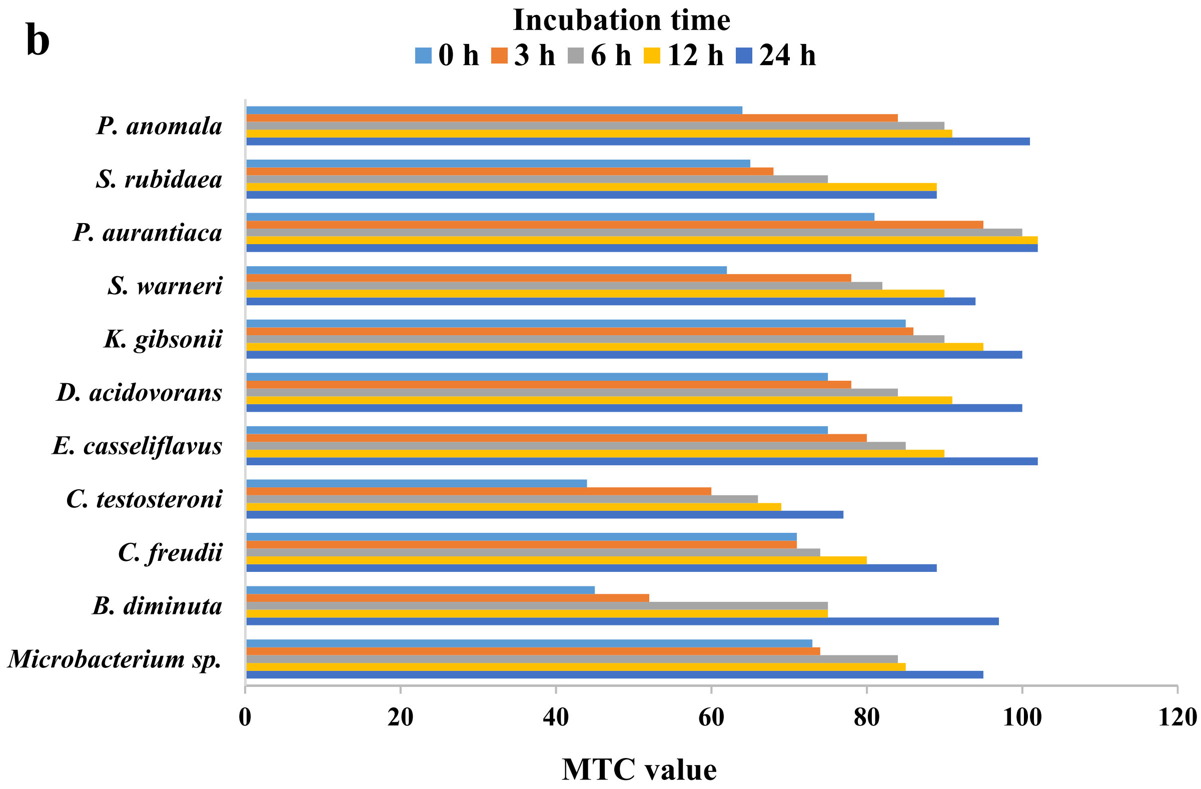
b
Incubation time
0 h
3 h
6 h
12 h
24 h
P. anomala
S. rubidaea
P. aurantiaca
S. warneri
K. gibsonii
D. acidovorans
E. casseliflavus
C. testosteroni
C. freudii
B. diminuta
Microbacterium sp.
0
20
40
60
80
100
120
MTC value

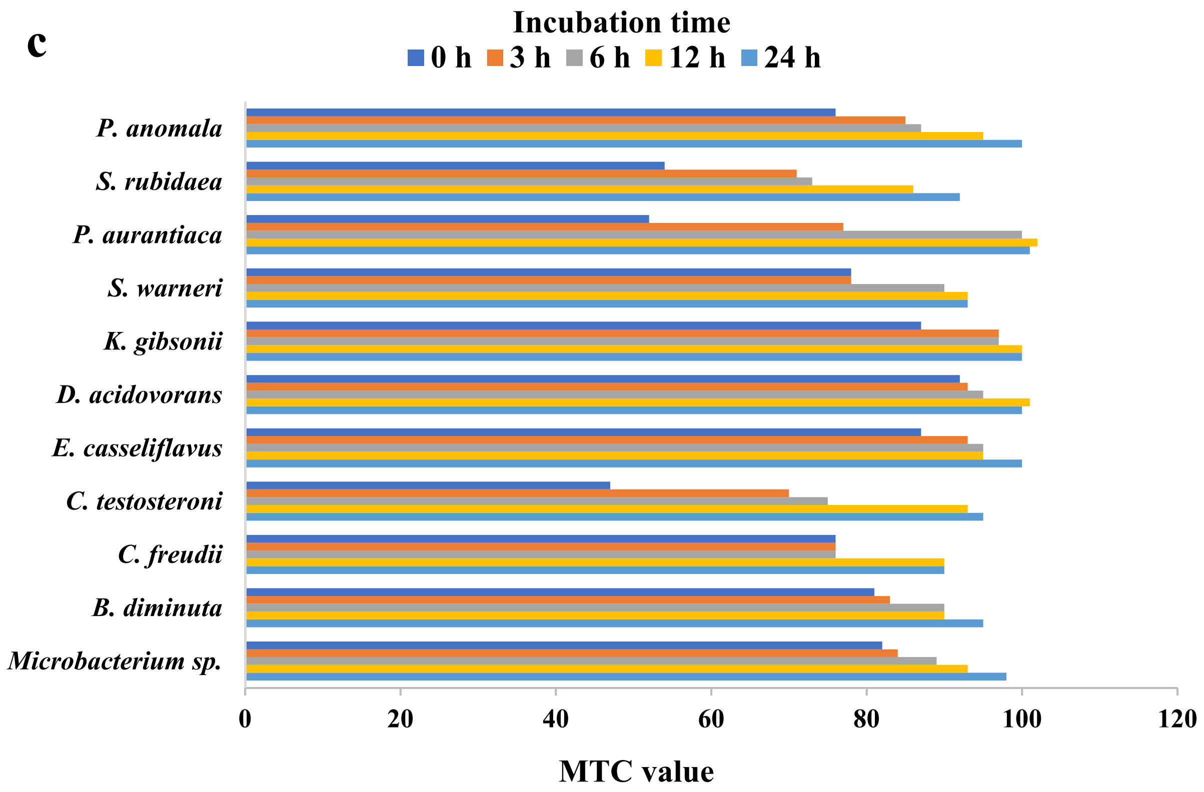

Figure S2. MARA species responses (MTC value) to *U. isabellina* cultures treated with test xenobiotics: a – 4-CP, b – NP, c – 4-*t*-OP.

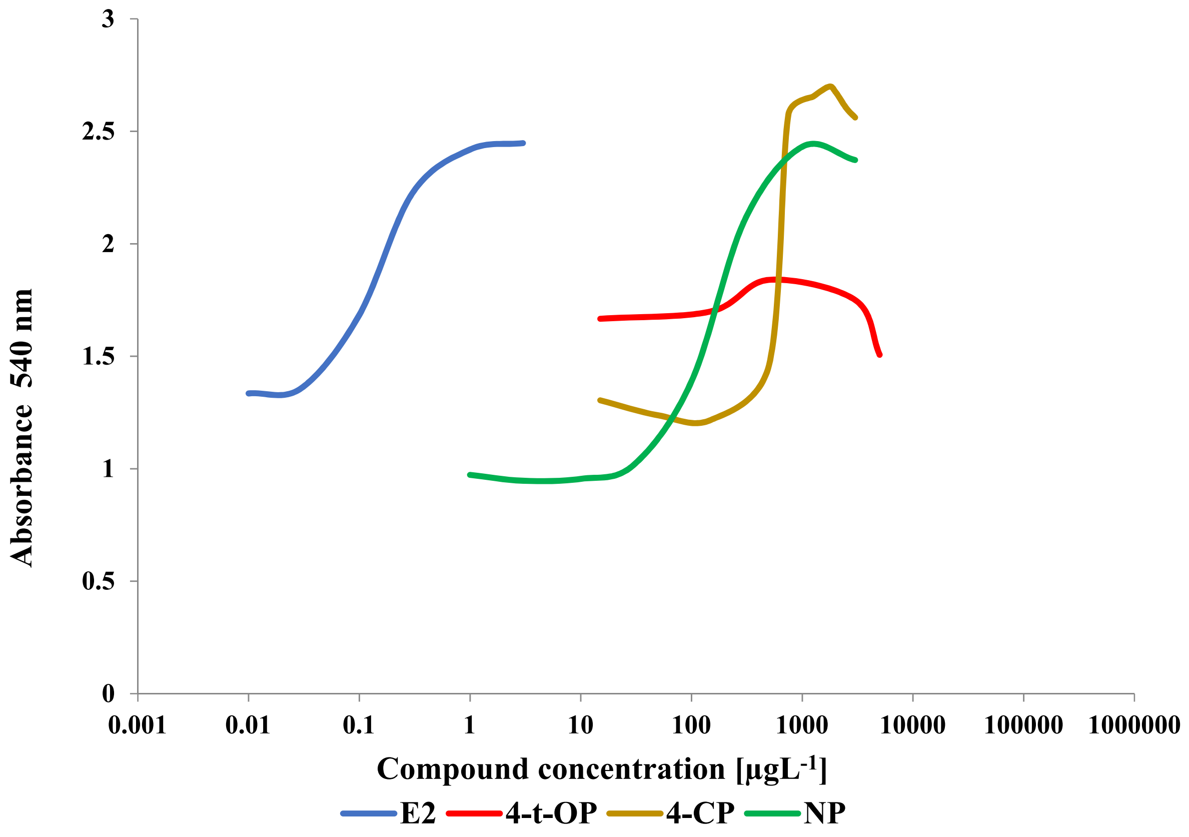

Figure S3. Oestrogenic activity of 4-CP, 4-*t*-OP and NP.

Table S4. Anti-androgenic activity of post-culture filtrates obtained after incubation of *U. isabellina* with NP or 4-CP (post-culture liquid concentration – 4%).

Time (h)	Absorbance of 4-CP (% of control, i.e. DHT)	Absorbance of 4-*t*-OP (% of control, i.e. DHT)
0	65.50 ± 1.52	75.04 ± 1.12
3	75.01 ± 0.90	83.20 ± 1.42
6	82.28 ± 1.23	85.18 ± 0.64
12	81.98 ± 0.56	87.90 ± 0.49
24	86.73 ± 0.70	93.72 ± 0.80

Table S5. Plant biological endpoints for testing the phytotoxicity of fungal post-culture filtrates supplemented with landfill leachate.

Landfill leachate (%)	Time (h)	Germination inhibition (PE%)	Root growth inhibition (PE%)	Germination index (%)
20	0	0 ± 0	32.5 ± 2.8	59.5 ± 2.0
	24	0 ± 0	39.7 ± 2.2	52.4 ± 3.0
	48	0 ± 0	49.6 ± 3.0	43.0 ± 2.9
	72	0 ± 0	29.3 ± 2.7	62.9 ± 3.6
	96	0 ± 0	20.6 ± 3.1	78.3 ± 2.8
40	0	10 ± 2	59.1 ± 3.3	40.6 ± 1.7
	24	0 ± 0	64.7 ± 2.8	35.2 ± 2.6
	48	0 ± 0	69.2 ± 3.4	30.8 ± 2.0
	72	0 ± 0	53.4 ± 3.8	44.6 ± 2.7
	96	0 ± 0	40.1 ± 2.5	59.7 ± 2.1
